# A head-to-head comparison of the adult EQ-5D-5L and youth EQ-5D-Y-5L in adolescents with idiopathic scoliosis

**DOI:** 10.1186/s41687-025-00842-z

**Published:** 2025-01-29

**Authors:** Joshua M. Bonsel, Charles M. M. Peeters, Max Reijman, Tim Dings, Joost P. H. J. Rutges, Diederik H. R. Kempen, Jan A. N. Verhaar, Gouke J. Bonsel

**Affiliations:** 1 Department of Orthopaedics and Sports Medicine, Erasmus MC, University Medical Center, Doctor Molewaterplein 40, 3015GD, Rotterdam, Room Nc-424, PO box 2040, Rotterdam, 3000CA The Netherlands; 2 Department of Orthopaedics, University Medical Center Groningen, Groningen, The Netherlands; 3 Department of Orthopaedics, Isala Hospital, Zwolle, The Netherlands; 4 Department of Orthopaedics, OLVG, Amsterdam, The Netherlands; 5 Department of Orthopaedics, Amsterdam University Medical Center, Amsterdam, The Netherlands; 6 EuroQol Research Foundation, Rotterdam, The Netherlands

**Keywords:** EQ-5D-5L, EQ-5D-Y-5L, Children, Adult, Youth, Quality of life, Equivalence, Longitudinal, Clinical registries, Adolescent idiopathic scoliosis

## Abstract

**Background:**

Multiple diseases, such as Adolescent Idiopathic Scoliosis (AIS), present at adolescent age and the impact on quality of life (QoL) prolongs into adulthood. For the EQ-5D, a commonly used instrument to measure QoL, the current guideline is ambiguous whether the youth or adult version is to be preferred at adolescent age. To assess which is most suitable, this study tested for equivalence along predefined criteria of the youth (EQ-5D-5L) and adult (EQ-5D-Y-5L) version in an adolescent population receiving bracing therapy for AIS.

**Methodology:**

107 adolescents were recruited from 4 scoliosis centers in the Netherlands between March 2022 and January 2023; they completed both EQ-5D’s and the SRS-22r (scoliosis-specific questionnaire). The following criteria were evaluated using the individual and sum of domains (level-sum-score (LSS)). Our primary criterion for non-equivalence of the EQ-5D’s was less than excellent (≤ 0.9) intra-individual agreement using Intraclass Correlation Coefficient (ICC) analysis for LSS and weighted (quadratic) kappa for domains. Secondary criteria were differences in ceiling using McNemar test; a different number of quantified hypotheses for construct validity achieved using the SRS-22r as comparator; differences in test-retest reliability by comparing ICC/kappa values using a Z-test.

**Results:**

Adolescents had a mean age of 14 years (range 12–18), and 78% were female. Ceiling was mostly comparable between EQ-5D’s, ranging from 78 to 81% for mobility and self-care, 52–54% for usual activities, and 31–36% for pain/discomfort. The EQ-5D-5L showed more ceiling (57%) compared to the EQ-5D-Y-5L (41%) on anxiety/depression (*p* = 0.006). Agreement between the EQ-5D’s did not meet our criterion for the LSS (ICC 0.79 (95% confidence interval 0.70–0.85)), and decreased further at the domain-level. Both EQ-5D’s achieved 5/7 validity hypotheses. Test-retest reliability was slightly better for EQ-5D-5L LSS (ICC 0.76 (0.64–0.84)) compared to EQ-5D-Y-5L LSS (ICC 0.69 (0.55–0.79)), although this was statistically insignificant (*p* = 0.284). This pattern was similar for most domains.

**Conclusions:**

The EQ-5D versions showed insufficient agreement, and cannot be considered fully equivalent. While they were similar in terms of validity and test-retest reliability, differences in score distribution were present. Taken together, we advise using the EQ-5D-5L to monitor the QoL in adolescent patients with AIS, as it avoids switching instruments and thus data discontinuities. Future studies should verify these findings in different patient groups and the general population.

**Supplementary Information:**

The online version contains supplementary material available at 10.1186/s41687-025-00842-z.

## Background

Health-Related Quality of Life (HRQoL) in children and adults, preferably self-reported, is recognized as an essential outcome parameter in medical practice and research. The EQ-5D is a widely used instrument to measure HRQoL in adults [1], and 2 versions are available in terms of the number of response levels: the 3-level (EQ-5D-3 L) and 5-level (EQ-5D-5L) version. A decade ago, a youth version was developed aimed at children from 8 to 11 years of age [2, 3]. The intended concept and general structure were the same as the adult version, while the wording and content were tailored towards children. Currently, the youth version of the EQ-5D is also available as 3-level (EQ-5D-Y-3 L) and 5-level (EQ-5D-Y-5L) version. Contemporary evidence has shown that the adult EQ-5D-5L (adult) has superior discriminatory power with less ceiling and a similar psychometric pattern as the EQ-5D-Y-5L (youth) [4–7]. Therefore, our study uses the 5-level versions.

Our research focused on the age-specificity of both versions. Specifically, our study tests the equivalence of the EQ-5D-5L and EQ-5D-Y-5L with data from Adolescent Idiopathic Scoliosis (AIS) patients who receive bracing treatment. Current guidelines from the EuroQol Research Foundation suggest the EQ-5D-Y self-report to be used in the younger age range (8–11 years) for its better comprehensibility [8]. In adolescents (12–18 years) neither version is preferred. Indirect evidence suggests that the EQ-5D-5L and EQ-5D-Y-5L perform equally well regarding validity, reliability, and responsiveness in this adolescent population [4, 9, 10]. Yet, head-to-head comparative evidence is absent. If the EQ-5D-5L and EQ-5D-Y-5L indeed are psychometrically similar (‘equivalent’) in adolescents, and otherwise comparable in practical application, this would imply the versions can be used interchangeably. If true, this would signify a preference for the EQ-5D-5L as it avoids the switching of versions at an age threshold in longitudinal applications. If the versions are not equivalent and the EQ-5D-Y-5L performs better in terms of alignment with the experience, language, and reflective abilities of adolescents, then this version should be preferred up to the age of 17.

AIS is the most common type of scoliosis; about 3 to 5 per 1000 children are estimated to develop AIS requiring treatment [11]. Although AIS patients are generally healthy apart from the deformity, the disease often decreases the quality of life through the experienced pain and social impact. Moreover, due to various treatment modalities such as bracing or surgery, AIS patients also face problems with self-image and mental health [12–14]. As the disease impact, the associated burden, and the side-effects of treatment inevitably prolong into adulthood, this population is a prime example to study the continuity of HRQoL instruments longitudinally.

In this study, we hypothesize that the EQ-5D versions are equivalent in this adolescent population regarding (1) intra-individual agreement, (2) distributional properties, in particular ceiling, (3) performance in validity tests, and (4) test-retest reliability. The criteria norms are discussed in the methods section.

## Methodology

### Study design

Questionnaires and other data were collected prospectively. This study was approved by the Medical Ethical Review Board from University Medical Center Groningen (reference number 202100536); study-site specific ethical approval of each participating center was also obtained. Although this study was not pre-registered, we developed a statistical plan before data collection was complete. This manuscript is written according to the Guidelines for Reporting Reliability and Agreement Studies and COSMIN reporting guideline for studies on measurement properties of Patient-Reported Outcome Measures (PROMs) [15, 16]. We aimed for at least 100 participants advised by the COSMIN guidelines.

### Participants

Consecutive patients from 4 scoliosis centers were included at the outpatient clinics between March 2022 and January 2023 if they met the following inclusion criteria: diagnosis of AIS, under active treatment with bracing, and age between 12 and 18 years. The diagnosis of AIS is made after other causes for (secondary) scoliosis have been excluded or are deemed unlikely. The disease severity is typically measured using the Cobb angle on spine radiographs. Patients receive bracing therapy generally for moderate curvatures and upwards, i.e., a Cobb angle > 20°, with the aim to prevent further curve progression and the need for spinal surgery [11, 17]. Patients were excluded who underwent surgery or inability to complete study questionnaires due to cognitive impairment or insufficient understanding of the Dutch language.

### Procedures

Eligible patients (and their parent/guardian) received oral and standardized written information on the study, and participants were required to provide consent conforming to Dutch law. Adolescents aged 12 to 16 give are required to provide consent independently in addition to their parents or guardian. From 17 and older, adolescents sign themselves. After obtaining signed informed consent, patients were sent a first link to a set of questionnaires in an electronic data-capture system (Castor). The first set of questionnaires included (1) various demographics, (2) the EQ-5D-5L (and EQ Visual Analogue Scale (VAS)), (3) the SRS-22r which has no defined age-limits, and (4) the EQ-5D-Y-5L (and EQ VAS). No missing data were allowed; however, one patient aborted the survey too early resulting in one missing value for the EQ VAS. The order of the EQ-5D versions was individually randomized. On top of these questionnaires, 75% of patients also filled out a novel Brace Questionnaire (BrQ) to assess its validity; the results have been recently published and are not discussed or used in this study [18]. To assess test-retest reliability, patients were sent a second link 7–14 days after completion of the first set of questionnaires.

### Questionnaires

#### Demographics

Obtained demographics included age, sex, education level, body mass index (BMI), menarche (if female) and Cobb angle at inclusion. In the Netherlands, education can be trichotomized into primary education (i.e., primary school), secondary education (i.e., preparatory vocational, secondary vocational education, preparatory general education, or preparatory university education), and tertiary education (i.e., higher professional education or university education) [19]. Secondary education is generally known as high school. We collapsed secondary and tertiary education in two groups: *practical education* which included preparatory vocational or secondary vocation education and *theoretical education* which included preparatory general and preparatory university education, and also higher professional and university education.

#### EQ-5D-5L and EQ-5D-Y-5L

The official Dutch translation of the five-level versions of the EQ-5D-5L and EQ-5D-Y-5L was used [20]. Both versions cover 5 domains (Mobility, Self-care, Usual activities, Pain/Discomfort, and Anxiety/Depression), and both have 5 response levels resulting in 3125 possible health states.

The EQ-5D-Y-5L differs from the EQ-5D-5L in the following: (1) ‘walking about’ is added as explanation to the domain header ‘Mobility’; (2) the domain header ‘Self-care’ is changed into ‘Looking after myself’; (3) child-relevant examples are listed after the domain header ’Usual activities’ (‘going to school, hobbies, sports, playing, doing things with family or friends’); (4) the domain header ‘Pain/Discomfort’ is changed into ‘Pain or other complaints’; (5) the domain header ‘Anxiety/Depression’ is changed into ‘Feeling worried, Sad or Unhappy’. The most obvious difference concerns (6) the response levels: supposedly more child-friendly terms for level 3 and 4 are used in the EQ-5D-Y-5L. (7) Also, the most extreme level 5 is formulated slightly different for the domains ‘Mobility’, ‘Self-care’ and ‘Daily activities’: the phrase ‘I am not able to’ is replaced with ‘I cannot’. The changes of the Y-version were the result of extensive qualitative and quantitative testing [2, 3]. The question texts (in Dutch) are included in Supplementary Material 1; the full versions can be requested from the EuroQol Research Foundation.

The EQ-5D-5L has country-specific preference-based value sets available (for both 3 L and 5L), that transforms each health state into an aggregate score, including the Netherlands [21]. For the EQ-5D-Y-5L currently only 3 L value sets are available, and 5L sets are on their way [22]. As the primary goal of our research is descriptive equivalence, and in view of the absence of valuation sets for the currently used EQ-5D-Y-5L version, we use the level sum score (LSS) to compare aggregate scores between the instrument versions. Using the LSS, the best possible score is 1 + 1 + 1 + 1 + 1 = 5, and the worst possible score is 5 + 5 + 5 + 5 + 5 = 25. This conforms to current practice in non-economic papers, including research into descriptive performance [23].

#### EQ VAS

The EQ VAS aims to measure overall quality of life, and is a combination between a traditional Numerical Rating Scale and a Visual Analogue Scale. It is presented vertically. At the top a label states ‘the best imaginable health’. The scale ranges from 0 (worst) to 100 (best), with ticks on the scale at each increment of 10. The youth version of the EQ VAS differs from the adult version in the following: (1) an informal version of the Dutch pronoun ‘you’ is used, and (2) the term ‘measuring scale’ is replaced by ‘line’.

#### SRS-22r

The SRS-22r is a commonly used AIS-specific questionnaire developed and validated for adolescents, which we used as the comparator/reference for validity analysis [12, 24, 25]. It covers the domains function, pain, self-image, mental health, and satisfaction/dissatisfaction with management. Each domain consists of 5 items except for satisfaction/dissatisfaction, which consists of 2 items. Domain and aggregate scores are calculated by averaging the item-scores for each domain, and all items, respectively; scores range from 1 to 5, where a higher score indicates a better outcome.

### Statistical analysis

#### General

In view of our research goal, the null hypothesis (to be rejected) is that the two EQ-5D versions are not equivalent, while the alternative hypothesis claims equivalence. Hence, equivalence is to be proven. To test for the equivalence of a new version or collection modality of HRQoL instruments in comparison to a default version several recommendations are available [26, 27]. This entails non-inferiority testing of the new version, which evaluates whether the new version is not worse than the default version. In our study, we test for true equivalence (rather than non-inferiority) as there is no default; in other words, either version may be better than the other. We derived our set of criteria from the above recommendations, taking the absence of a default into consideration.

The primary criterion is head-to-head (intra-individual) agreement of ≥ 0.91 expressed by Intraclass Correlation Coefficients (ICC) for aggregate scores and kappa values for domains, conform the recommendations for application of PROMs at the individual level. Of note, for application at the group level, recommendations are more lenient and ICC and kappa values of ≥ 0.7 and ≥ 0.8 are considered acceptable, respectively. Three secondary psychometric criteria were: distributional properties (lack of ceiling in particular), validity, and test-retest reliability. In the context of longitudinal use of EQ-5D in registries covering adolescent and adult age, test-retest reliability has specific relevance. If the versions are equivalent based on the primary criterion, and are similar in practical features, we conclude that they are interchangeable. If the EQ-5D versions are not equivalent, we will prefer the version with the best psychometric performance on secondary criteria where test-retest reliability has extra weight.

For further statistical testing of strength of association, ICC, kappa and Spearman rank correlation analysis were used. ICC and kappa coefficients were interpreted as follows: poor (≤ 0.39), fair (0.40–0.59), good (0.60–0.74), and excellent (0.75-1.00) reliability [28]. Spearman rank coefficients (rho) were interpreted as: negligible (≤ 0.10), weak (0.11–0.39), moderate (0.40–0.69), strong (0.70–0.89), and very strong (≥ 0.90) correlation [29].

Below we provide details on the statistical analysis. All analyses were performed in R version 4.3.1 [30]. Where appropriate 95% confidence intervals (95% CIs) were reported, and a p-value < 0.05 was considered significant. R packages used are included in Supplementary Material 2.

#### Sample description

Sample characteristics were summarized, and conventional descriptive statistics for the EQ-5D-5L, EQ-5D-Y-5L, and SRS-22r responses were calculated. Aggregate scores between EQ-5D versions were compared using the Wilcoxon signed-rank test, while domains were compared using the Bowker’s test for symmetry.

#### Distributional characteristics: ceiling and floor effects

The proportion of patients reporting ‘no problems’ (ceiling) and ‘extreme problems’ (floor) for the LSS and each domain, were compared between the EQ-5D versions using the McNemar test. For reference, these procedures were also conducted for the EQ-VAS and the SRS-22r. Overall, we expected relatively high ceiling and any significant difference between EQ-5D versions was considered potentially relevant.

#### Intra-individual agreement

ICCs based on single measurement, absolute-agreement, two-way random effects model were calculated for the LSS of the EQ-5D versions [31]. An ICC absolute-agreement was selected for all comparisons, as systematic differences are also relevant in the overall appraisal of QoL. ICC absolute-agreement typically results in lower ICC estimates compared to ICC consistency, which excludes systematic differences. Weighted (quadratic) kappa values were calculated for domains. A relevant disagreement was defined as an ICC or kappa ≤ 0.90, as described above. If indeed intra-individual agreement was less than hypothesized, we explored the observed disagreement with Bland-Altman plots [32]. ICC’s and kappa are reliability parameters which relate the measurement error to the variation in the studied population, while Bland-Altman plots provide specific insights into the measurement error component. The Limits of Agreement (LOA), which were set at 95%, describe the size of measurement error between EQ-5D versions [33]. The dispersion of datapoints illustrate whether measurement error is random or systematic in nature. In case of the latter, future work may investigate the adjustability of this variation. Difference scores were assessed graphically and found to be roughly normaliy distributed, hence no data transformation was applied. Similar procedures were applied to the EQ VAS as reference.

#### Convergent validity

The strength of association using Spearman rank correlation was established between the EQ-5D-5L and the SRS-22r, and the EQ-5D-Y-5L and the SRS-22r, respectively. The COSMIN guidelines states that 75% of hypotheses should be met to assume validity. Associations were established between total scores, between similar domains (convergent validity, expectation: rho≤-0.40) and between conceptually unrelated domains (convergent validity, rho*≥*-0.39), based on previous literature [4, 9, 10]. We expected only negative associations given the EQ-5D is the only questionnaire for which lower scores reflect better health. We expected rho≤-0.40 for the comparison of EQ-5D self-care to SRS-22r function, EQ-5D pain to SRS-22r pain, EQ-5D anxiety/depression to SRS-22r self-image and EQ-5D anxiety/depression to SRS-22r mental health. We expected rho*≥*-0.39 for the comparison of EQ-5D mobility to SRS-22r function and EQ-5D usual activities to SRS-22r function. Finally, we inspected whether either questionnaire in general outperformed the other in terms of validity, considering a difference in number of thresholds achieved of 1 or more to be relevant.

#### Test-retest reliability

Using the same approach as under intra-individual agreement, ICCs and kappa values were calculated for the LSS and domains between the first and second measurements, for the EQ-5D-5L and EQ-5D-Y-5L separately. We applied the same thresholds for and expected test-retest reliability to exceed ≥ 0.91 for both EQ-5D versions. To evaluate differences in test-retest reliability among EQ-5D versions, we applied Fisher’s r-to-Z transformation to the coefficients and used a Z-test (Steiger’s) for dependent groups to determine statistical significance [34, 35]. Similarly, Bland-Altman plots were used to illustrate the measurement error from first to second measurement.

#### Sensitivity analysis

To check the robustness of the findings regarding *intra-individual agreement* and *test-retest reliability* in particular, we re-ran these analyses within known subgroups which reflect more vs. less severe disease based on previous literature [4, 9, 10]. ICCs and kappa values were recalculated in the following subgroups: a Cobb angle ≥ 30 vs. <30; SRS-22r sum-score best 50% vs. worst 50%; practical vs. theoretical education; age oldest 50% vs. youngest 50%. Due to the small number of children who were still in primary school (*n* = 8), these were not used in the comparison according to education.

## Results

Out of 175 eligible patients with AIS undergoing brace treatment, 107 provided informed consent and completed the first survey. Seventy-eight (75%) responded to the second survey at an average follow-up of 27 days (Standard Deviation (SD) 16, range 9–73). Patients were included at a mean age of 14 years (SD 1.4, range 12–18), and 83 (78%) were female (Table 1).

**Table 1:** Characteristics of study population

Total sample, *n* = 107	
Age in years, mean (SD)	14.3 (1.4)
Female, n (%)	83 (78)
Highest completed education, n (%)	
Primary education	8 (8)
Practical education	42 (40)
Theoretical education	57 (52)
Body mass index (kg/m2), mean (SD)	18.0 (2.6)
Menarche (if female, *n* = 83), n (%)	62 (75)
Cobb angle at inclusion*, n (%)	
≤30	46 (43)
>30	60 (57)

A higher Cobb angle indicates more severe scoliosis

*Data is missing from 1 patient

**Fig. 1:**
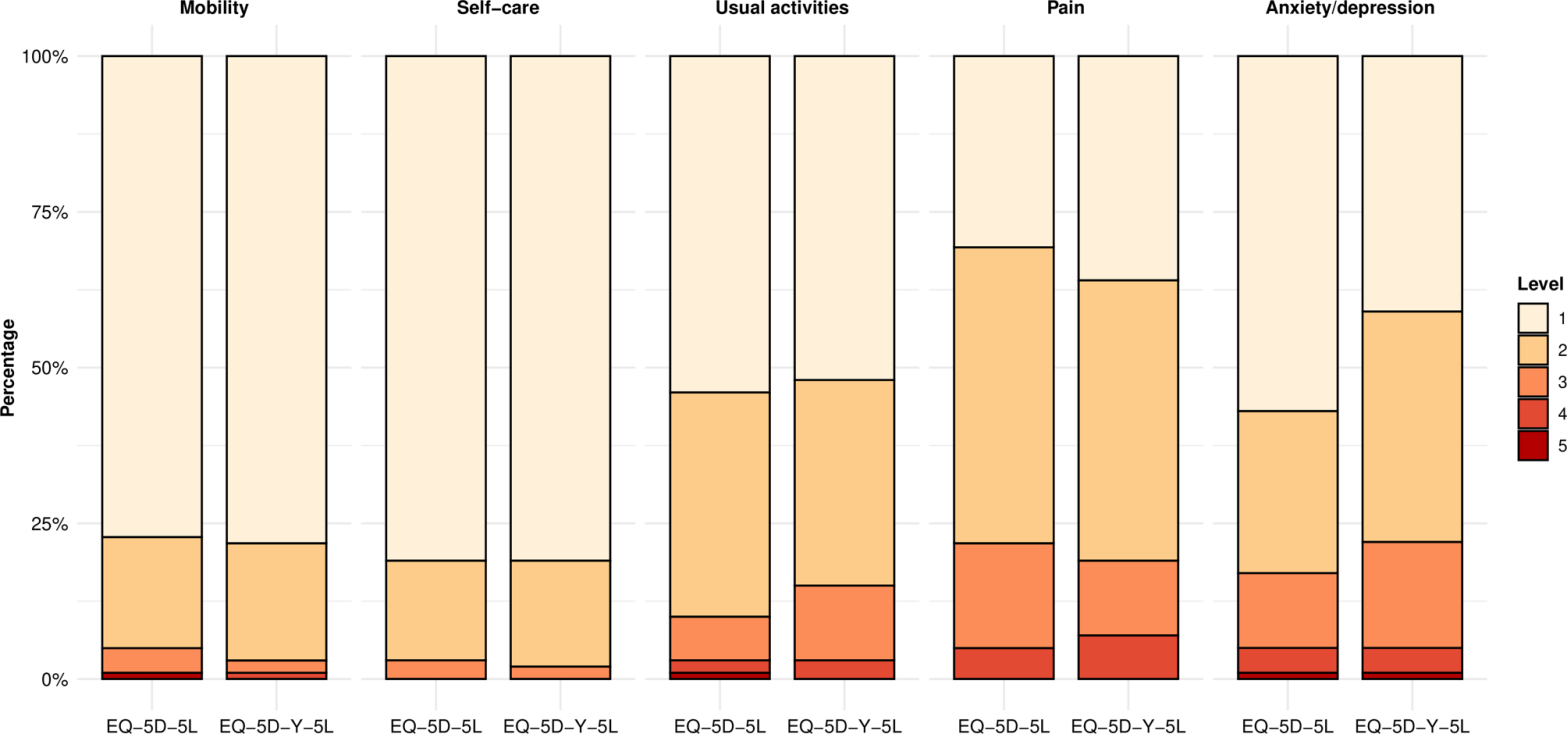
Distribution of the domain responses of the EQ-5D versions

The sample was relatively healthy, with high (low for LSS) average scores on all questionnaires (Table 2A, Fig. 1). The EQ-5D’s were similar with regard to aggregate scores: the median LSS was 7 (Interquartile Range (IQR) 6–9) for both the EQ-5D-5L and EQ-5D-Y-5L (*p* = 0.243). At the domain level on both EQ-5D’s, mobility and self-care were rated slightly better compared to usual activities, pain, and anxiety/depression. Median values of domain scores were also similar between EQ-5D’s. The median value for the aggregate SRS-22r score was 4.0 (IQR 3.5–4.4). Corresponding domains in SRS-22r and EQ-5D tended to produce a similar distributional pattern (Table 2B).

**Table 2A:** Descriptive statistics of EQ-5D versions

	EQ-5D-5L			EQ-5D-Y-5L			*p*-value (diff. in median)**	*p*-value (diff. in ceiling)***
	Median (IQR)	Range	Ceiling, *n* (%)	Median (IQR)	Range	Ceiling, *n* (%)		
*Aggregate*								
LSS	7 (6–9)	5–18	19 (18)	7 (6–9)	5–17	14 (13)	0.243	0.359
VAS*	87 (70–95)	42–100	15 (14)	85 (73–94)	45–100	13 (13)	0.785	1.000
*Domain*								
Mobility	1 (1–1)	1–5	83 (78)	1 (1–1)	1–4	84 (79)	0.795	1.000
Self-care	1 (1–1)	1–3	87 (81)	1 (1–1)	1–3	87 (81)	0.753	1.000
Usual act.	1 (1–2)	1–5	58 (54)	1 (1–2)	1–4	56 (52)	0.830	0.864
Pain/disc.	2 (1–2)	1–4	33 (31)	2 (1–2)	1–4	39 (36)	0.624	0.327
Anx./depr.	1 (1–2)	1–5	61 (57)	2 (1–2)	1–5	44 (41)	0.267	0.006

Ceiling effects were defined as the best score attainable. For the LSS and domain scores a lower score indicates better health, while for the SRS-22r and VAS a higher score indicates better health

LSS = level-sum-score; VAS = Visual Analogue Scale; diff. = difference; disc.=discomfort; anx.=anxiety; depr.=depression; IQR = Interquartile Range

*Data of the VAS (EQ-5D-Y-5L) is missing in 1 patient

**For aggregate scores the Wilcoxon signed-rank test was used, while for domain scores the Bowker test was used

***For all comparisons the McNemar test was used

**Table 2B:** Descriptive statistics of SRS-22r

	Median (IQR)	Range	Ceiling, *n* (%)
*Aggregate*			
Sum-score	4.0 (3.5–4.4)	2.2–4.8	0
*Domain*			
Function	4.4 (4.0–4.8)	2.8–5.0	16 (15)
Pain	4.2 (3.8–4.5)	1.4–5.0	9 (8)
Self-image	3.6 (3.0–4.1)	1.6–5.0	2 (2)
Mental health	3.8 (3.1–4.2)	1.0–5.0	3 (3)
Satisfaction with treatment	4.0 (3.5–4.5)	2.0–5.0	15 (14)

### Ceiling and floor

Both EQ-5D versions produced no floor regarding aggregate scores and max. 1% for domains. Ceiling was prominent: with regard to the LSS, the ceiling was slightly larger for the EQ-5D-5L (18%) compared to the EQ-5D-Y-5L (13%), although this did not differ significantly (*p* = 0.359). Ceiling was about similar for most domains of EQ-5D versions, and did not differ signficantly. The highest ceiling was observed for mobility (78% and 79%, for EQ-5D-5L and EQ-5D-Y-5L, respectively) and self-care (81% and 81%), and the lowest for pain (31% and 36%); usual activities was in-between (54% and 52%). The ceiling of the anxiety/depression domain was significantly higher for EQ-5D-5L (57%) compared to EQ-5D-Y-5L (41%) (*p* = 0.006).

### Intra-individual agreement

The agreement (ICC) between EQ-5D’s was 0.79 (95% CI 0.70–0.85) for LSS and 0.80 (95% CI 0.72–0.86) for VAS (Table 3). At the domain level, kappa values were smaller; they were highest for self-care and pain/discomfort, and lowest for usual activities and anxiety/depression. All ICC/kappa values were lower than our predefined threshold of ≥ 0.91.

**Table 3:** Agreement between EQ-5D versions

	Predefined hypothesis	ICC (95% CI)
*Aggregate*		
VAS	N/A	0.80 (0.72–0.86)
LSS	≥ 0.91	0.79 (0.70–0.85)

		**Kappa (95% CI)**
*Domain*		
Mobility	≥ 0.91	0.62 (0.38–0.86)
Self-care	≥ 0.91	0.76 (0.58–0.94)
Usual act.	≥ 0.91	0.48 (0.31–0.65)
Pain	≥ 0.91	0.69 (0.56–0.81)
Anx./depr.	≥ 0.91	0.60 (0.44–0.76)

ICC’s were calculated for the aggregrate scores, between the EQ-5D-A and the EQ-5D-Y. Kappa analysis was used to assess agreement for domains. None of the predefined hypotheses were met

LSS = level-sum-score; VAS = Visual Analogue Scale; N/A = not applicable; ICC = Intraclass Correlation Coefficient; 95% CI = 95% confidence interval

**Fig. 2:**
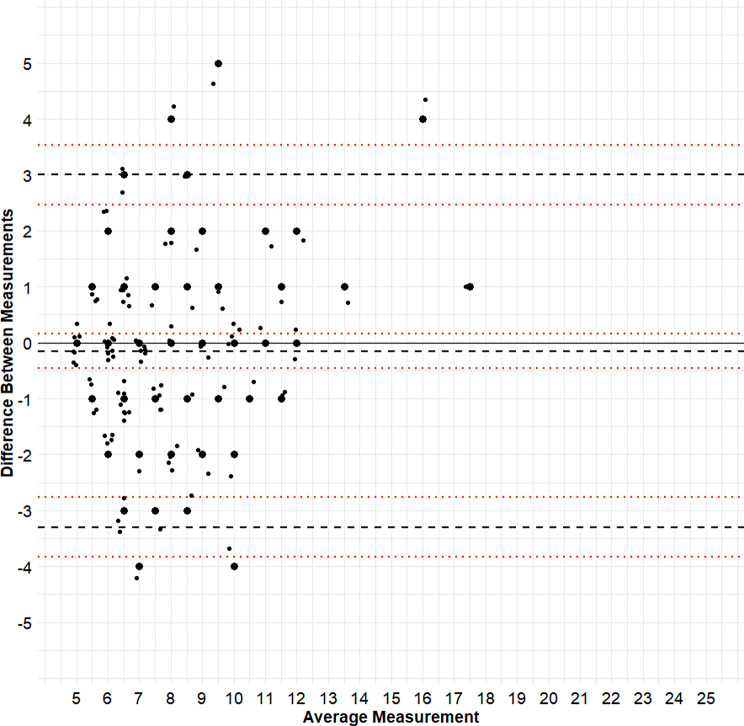
Bland-Altman plot for the LSS of the EQ-5D versions. The y-axis depicts the difference between the intra-individual measurement of the EQ-5D-5L and EQ-5D-Y-5L. The x-axis depicts the average of these two measurements. The dashed lines indicate the mean difference between EQ-5D versions and 95% limits of agreement. The red dotted lines represent the 95% confidence intervals for these estimates

**Fig. 3:**
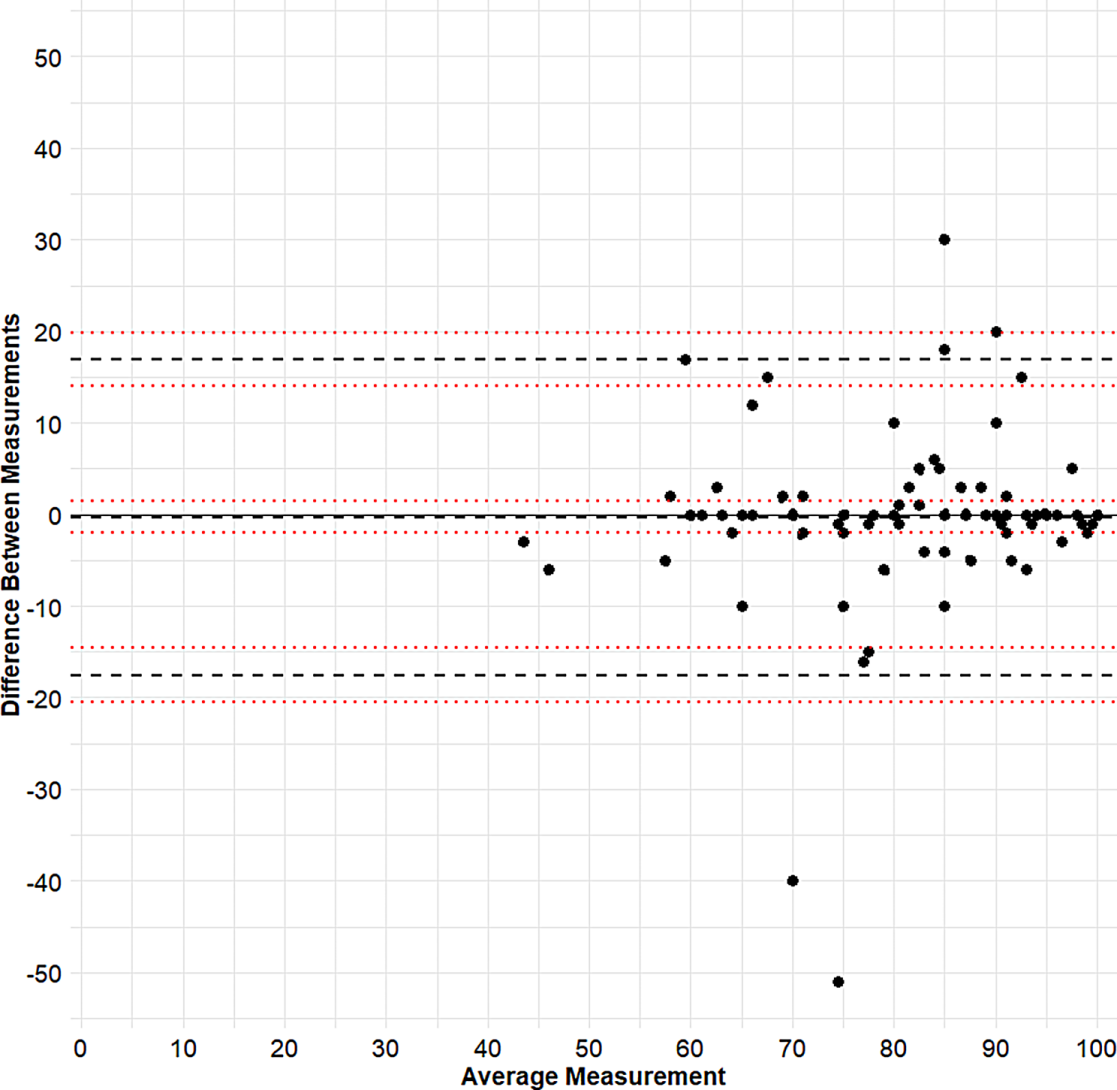
Bland-Altman plot for the VAS of the EQ-5D versions. The y-axis depicts the difference between the intra-individual measurement of the VAS obtained with the EQ-5D-5L and the VAS obtained with the EQ-5D-Y-5L. The x-axis depicts the average of these two measurements. The dashed lines indicate the mean difference between VAS versions and 95% limits of agreement. The red dotted lines represent the 95% confidence intervals for these estimates

Bland-Altman plots were created to gain insights into the measurement error between the EQ-5D versions (Figs. 2 and 3). For the LSS, the mean difference was − 0.15 (95% CI -0.46–0.16). The upper LOA was 3.00 (95% CI 2.47–3.53) and the lower LOA was − 3.30 (95% CI -3.82 – -2.77). In other words, 95% of differences between the LSS of EQ-5D’s fall between approximately − 3 and + 3. For the VAS, the mean difference was 0.29 (95% CI -1.99–1.40), upper LOA 16.94 (95% CI 14.00–18.87), lower LOA − 17.52 (95% CI -20.45 – -14.59). Overall, the plots suggested that disagreement was largely due to random variation, for both the LSS and VAS scores.

### Convergent validity

The pre-defined hypotheses with regard to validity were met for 5 out of 7 hypotheses pertaining to the LSS or domains, for both the EQ-5D-5L and EQ-5D-Y-5L (Table 4).

**Table 4:** Convergent validity of EQ-5D versions

		EQ-5D-5L	EQ-5D-Y-5L
	Predefined hypothesis	Rho (95% CI)	Rho (95% CI)
*Aggregrate*			
EQ VAS vs. SRS sum-score	N/A	0.57 (0.40–0.68)	0.52 (0.35–0.65
EQ-5D LSS vs. SRS sum-score	≤-0.40	-0.71* (-0.58 – -0.80)	-0.68* (-0.54 – -0.78)
EQ-5D LSS vs. EQ VAS	≤-0.40	-0.57* (-0.40 – -0.68)	-0.48* (-0.30 – -0.62)
*Domain*			
EQ-5D mobility vs. SRS function	≥-0.39	-0.36* (-0.18 – -0.52)	-0.25* (-0.07 – -0.43)
EQ-5D self-care vs. SRS function	≤-0.40	-0.16 (0.04 – -0.34)	-0.08 (0.12 – -0.27)
EQ-5D usual act. vs. SRS function	≥-0.39	-0.61 (-0.46 – -0.73)	-0.44 (-0.27 – -0.59)
EQ-5D pain vs. SRS pain	≤-0.40	-0.64* (-0.50 – -0.75)	-0.61* (-0.46 – -0.73)
EQ-5D anx./depr vs. SRS self-image	≤-0.40	-0.49* (-0.32 – -0.63)	-0.54* (-0.39 – -0.67)
EQ-5D anx./depr vs. SRS mental health	≤-0.40	-0.63* (-0.48 – -0.74)	-0.65* (-0.51 – -0.76)

Spearman rank correlations were calculated between the aggregate and domain scores. A higher EQ-5D domain/aggregate score indicates worse health, while a higher EQ VAS and SRS-22r domain/aggregate score indicates better health

LSS = level-sum-score; VAS = Visual Analogue Scale; N/A = not applicable; 95% CI = 95% confidence interval

*indicates if the predefined hypotheses was met

### Test-retest reliability

ICCs were 0.76 (95% CI 0.64–0.84) for the EQ-5D-5L LSS and 0.69 (95% CI 0.55–0.79) for the EQ-5D-Y-5L; see Table 5A. Test-retest reliability was lower at the domain-level, with the lowest kappa value observed for the self-care domain (EQ-5D-5L: 0.29 (95% CI 0.03–0.56), EQ-5D-Y-5L: 0.19 (95% CI -0.06–0.43)) and the highest for the anxiety/depression domain (EQ-5D-5L: 0.67 (95% CI 0.48–0.85), EQ-5D-Y-5L: 0.69 (95% CI 0.56–0.82)). Slightly higher point-estimates were generally observed for aggregate and domain scores of the EQ-5D-5L as compared to EQ-5D-Y-5L, however, these were not statistically significantly different. The Bland-Altman plots suggested that the difference between baseline and second measurement were mainly attributable to random variation rather than due to true change (Supplementary Material 3 – Fig. 1 to 4). For reference, Table 5B depicts the ICCs of the SRS-22r.

**Table 5A:** Test-retest reliability of EQ-5D versions

	Predefined hypothesis	EQ-5D-5L	EQ-5D-Y-5L	*p*-value (diff. in ICC/kappa)*
*Aggregrate*		**ICC (95% CI)**	**ICC (95% CI)**	
VAS	N/A	0.45 (0.26–0.61)	0.50 (0.32–0.65)	0.621
LSS	≥ 0.91	0.76 (0.64–0.84)	0.69 (0.55–0.79)	0.284
		**Kappa (95% CI)**	**Kappa (95% CI)**	
*Domain*				
Mobility	≥ 0.91	0.40 (0.19–0.60)	0.50 (0.31–0.68)	0.376
Self-care	≥ 0.91	0.29 (0.03–0.56)	0.19 (-0.06–0.43)	0.442
Usual act.	≥ 0.91	0.64 (0.46–0.81)	0.51 (0.32–0.70)	0.156
Pain	≥ 0.91	0.66 (0.53–0.79)	0.58 (0.41–0.75)	0.360
Anx./depr.	≥ 0.91	0.67 (0.48–0.85)	0.69 (0.56–0.82)	0.732

ICC’s and kappa values were calculated for the aggregate and domain scores, between the first and second measurement at least 7 days later (average 27 days later). None of the predefined hypotheses were met

LSS = level-sum-score; VAS = Visual Analogue Scale; ICC = Intraclass Correlation Coefficient; diff. = difference; N/A = not applicable; 95% CI = 95% confidence interval

*To compare ICC and kappa values, a Fisher’s r-to-Z transformation was applied and a Z-test (Steiger) was used to determine statistical significance

**Table 5B:** Test-retest reliability of SRS-22r

	ICC (95% CI)
Aggregrate	
Sum-score	0.87 (0.80–0.92)
Domain	
Function	0.70 (0.61–0.83)
Pain	0.76 (0.65–0.84)
Self-image	0.84 (0.76–0.90)
Mental health	0.79 (0.69–0.86)
Satisfaction with treatment	0.67 (0.53–0.78)

### Sensitivity analysis

The intra-individual agreement was relatively higher in subgroups with more severe scoliosis as defined by the SRS-22r or Cobb angle for both versions (Supplementary Material 4 - Tables 1–8). In contrast, agreement was lower in patients less affected by scoliosis. The subgroups education and age appeared to not affect the agreement. Test-retest reliability was similar according to Cobb angle, education and age, while better reliability was observed in patients with worse SRS-22r scores. The differences in points-estimates between the EQ-5D-5L and EQ-5D-Y-5L generally persisted (Supplementary Material 4 - Tables 9–16).

## Discussion

### Main findings

In this study, we compared the EQ-5D-5L and EQ-5D-Y-5L in a sample of AIS patients treated with a brace. Intra-individual agreement across versions was found to be excellent for the LSS (ICC 0.79 (95% CI 0.70–0.85)), however, did not meet our primary criterion for equivalence. Agreement further dropped at the domain level, in particular for *mobility*, *usual activities*, and *anxiety/depression*. Regarding psychometric properties, ceiling was comparable for most domains and the LSS, except for the *anxiety/depression* domain which showed sigifiicantly more ceiling for the EQ-5D-5L (57%) compared to the EQ-5D-Y-5L (41%). This may be attributed to the different wording of both question and response. Both the EQ-5D-5L and EQ-5D-Y-5L demonstrated comparable validity, achieving 5 out of 7 hypotheses (close to the commonly used 75% threshold). With regard to test-retest reliability, point-estimates were slightly higher for the EQ-5D-5L (LSS 0.76 (95% CI 0.64–0.84)) as compared to the EQ-5D-Y-5L (LSS 0.69 (0.55–0.79)), although these differences did not reach significance. As secondary psychometric criteria overall were roughly similar between EQ-5D versions, we think that in the context of patient monitoring from adolescence to adulthood the EQ-5D-5L is the preferred instrument. This avoids potential data discontinuities resulting from switching between versions and hence facilitates longitudinal follow-up from adolescence into adulthood.

### Comparison with other literature

This study is based on adopted criteria, which can greatly influence the judgement of determining (non-)equivalence. We chose to require intra-individual agreement (and test-retest reliability) to achieve strict thresholds, as we believe using EQ-5D versions interchangeably requires the instruments to align very strongly. However, for the purpose of larger group comparisons, more lenient thresholds may be used, as described in the methods section. Both EQ-5D versions showed acceptable intra-individual agreement and test-retest reliability for the LSS using these thresholds, but not at the domain level. Although no studies are available to compare the level of intra-individual agreement, test-retest reliability findings of both EQ-5Ds were in line with previous studies [9, 10]. In retrospect, it was unlikely for the reliability of EQ-5Ds to achieve the strict threshold we applied.

Lack of reliability of the EQ-5Ds was mostly attributable to random error, presumably because each domain includes only one question [36]. For longitudinal follow-up of patients, higher test-retest reliability translates into being able to more precisely capture a given health state. A more precise measurement of a given health state is desirable for research purposes, but also for clinical applications. The EQ-5D (and other PROMs) are increasingly used to guide clinical decision making, e.g., to determine whether surgical recovery is abnormal and potentially requires intervention [37]. Inaccuracies in the measurement of the health state may result in insufficient capacity to discriminate between patients with an abnormal recovery from patients with a normal/sufficient recovery. Given these potential clinical implications, it is imaginable that the version with a trend of higher test-retest reliability estimates may be the preferred option in this adolescent AIS population, i.e., the EQ-5D-5L.

As both EQ-5D versions have the same number of response levels, three underlying mechanisms may explain the disagreement between instrument versions for the domains *mobility*, *usual activities*, and *anxiety/depression*. Firstly, due to different wording of the question these domains cover a different underlying idea/concept. Secondly, they cover the same idea/concept, but the average distribution of scores is shifted lower or higher in general. Thirdly, due to different wording of the five severity labels, the distribution of the numbers (response) is different. In the first case one expects, if tested against an external anchor such as the SRS-22r, that the ranking of the responses of both versions is different. As this was not the case, the first explanation seems unlikely. In the second and third mechanism, one would expect the ranking to be similar despite a different use of the scale (distribution). In view of the fairly limited textual adaptations of the youth version, the results seem to match these explanations. The second mechanism is exemplified by the higher ceiling for *anxiety/depression* for the EQ-5D-5L compared to the EQ-5D-Y-5L. The EQ-5D-5L describes this domain as “fear/sadness”, while the EQ-5D-Y-5L describes it as “worrying, sadness or unhappiness”. In this situation, the underlying response scale may be shifted upwards in a constant fashion, hence patients use extreme values (ceiling) more often while correlation between measures remains relatively preserved. The third mechanism is expected to apply to the *mobility* and *usual activities* domains.

The EQ-5D-5L and EQ-5D-Y-5L demonstrated comparable validity. The validity findings were generally compatible with previous studies, and were close to the currently accepted 75% guideline for demonstrating validity [9, 10, 38, 39]. The LSS and SRS-22r sum scores were strongly correlated, suggesting that the EQ-5D is able to capture the relevant disease burden and HRQoL of AIS patients treated with a brace. We found insufficient association between the EQ-5D domain self-care and SRS-22r function domain (rho − 0.16 (EQ-5D-5L) and − 0.08 (EQ-5D-Y-5L) instead of ≥-0.40). A higher than expected association was found between the EQ-5D domain usual activities and the SRS-22r function domain (rho − 0.61 (EQ-5D-5L) and − 0.44 (EQ-5D-Y-5L) instead of ≤-0.39) [9]. The SRS-22r function domain focuses on the level of activity, on limitations in doing things around the house, financial difficulties due to AIS, and limits in going out with friends [12, 24]. These (mild) differences between our study and previous papers may be attributable to differences between samples: only 11% of the sample in the study by Adobor et al. was undergoing brace treatment at the time of filling out the questionnaire, and a larger percentage had surgery (39%) or were scheduled for surgery (30%), hence representing a population with more severe scoliosis. It is imaginable that a patient with more severe scoliosis have increased problems with *self-care* thus correlating more strongly with the SRS-22r function domain.

### Strengths and limitations

The present study had some limitations. Firstly, a sample size of 107 can be considered small, however, it does meet the current COSMIN criteria and the homogeneity of the sample permits careful testing [15]. Secondly, we did not include a question on experienced health change at the second measurement. Generally, excluding patients who report a change in health may benefit test-retest reliability. However, this would have added to the questionnaire burden already consisting of two close to identical questionnaires and a comparator. Also, we think a health change is unlikely in these rather healthy persons, as they were approached after they had already initiated bracing therapy and were still required to wear their brace until at least the subsequent visit which in general is 6 months later. Thirdly, as the study population was rather healthy, data was skewed. This affected the size of the kappa, resulting in lower values than would be expected for the observed absolute agreement. Finally, the current study is performed in a selected AIS population undergoing bracing treatment, and is inevitably not generalizable to all AIS patients. While AIS patients show a wide range of symptoms, specific patient groups may exist where the instrument versions show larger differences, or no difference at all.

## Conclusion

This is the first head-to-head comparison of the EQ-5D-5L and EQ-5D-Y-5L in an adolescent AIS population treated with a brace, using a strict testing format to reject or establish equivalence. The EQ-5D versions show insufficient intra-individual agreement and cannot be considered fully equivalent, and thus and cannot be used interchangeably. Although they were roughly similar in terms of validity and test-retest reliability, specific differences in score distribution were present. If longitudinal measurement of HRQoL from adolescence into adulthood is foreseen, and we think the EQ-5D-5L is the preferred choice with the added benefit that potential data discontinuities are avoided. Future studies should verify if this finding holds in different patient groups and the general population.

## Electronic supplementary material

Below is the link to the electronic supplementary material.

Supplementary Material 1

Supplementary Material 2

Supplementary Material 3

Supplementary Material 4

## Data Availability

The currently used dataset have been archived in a data repository (link: 10.34894/PDJZXH) and are available upon reasonable request, after approval by the author team. As the data are sensitive in nature, there are restrictions in place with regard to the availability of the data. Codes used to conduct the analyses are obtainable from the corresponding author.

## Abbreviations

AIS: Adolescent Idiopathic Scoliosis
BMI: Body mass index
BrQ: Brace Questionnaire
HRQoL: Health-Related Quality of Life
ICC: Intraclass Correlation Coefficient
LSS: Level sum score
IQR: Interquartile Range
SD: Standard Deviation
VAS: Visual Analogue Scale
