# Supplementary material for: A head-to-head comparison of the adult EQ-5D-5L and youth EQ-5D-Y-5L in adolescents with idiopathic scoliosis
Source: J Patient Rep Outcomes. 2025 Jan 29;9:13. doi: 10.1186/s41687-025-00842-z (PMC11780234; doi:10.1186/s41687-025-00842-z)
Supplement: Supplementary file 1 — Supplementary Material 1 [file 41687_2025_842_MOESM1_ESM.docx]

**Supplemental Material 1: EQ-5D-5L and EQ-5D-Y-5L questions**

**EQ-5D-5L (adult) version**

| **Domain header** | **Levels** |
| --- | --- |
| Mobiliteit | Ik heb ***geen*** problemen met lopen |
| Mobiliteit | Ik heb ***een beetje*** problemen met lopen |
| Mobiliteit | Ik heb ***matige*** problemen met lopen |
| Mobiliteit | Ik heb ***ernstige*** problemen met lopen |
| Mobiliteit | Ik ben ***niet*** in staat om te lopen |
| Zelfzorg | Ik heb ***geen*** problemen met mijzelf wassen of aankleden |
| Zelfzorg | Ik heb ***een beetje*** problemen met mijzelf wassen of aankleden |
| Zelfzorg | Ik heb ***matige*** problemen met mijzelf wassen of aankleden |
| Zelfzorg | Ik heb ***ernstige*** problemen met mijzelf wassen of aankleden |
| Zelfzorg | Ik ben ***niet*** in staat mijzelf te wassen of aan te kleden |
| Dagelijkse activiteit (bijv. werk, studie, huishouden, gezins- en vrijetijdsactiviteiten) | Ik heb ***geen*** problemen met mijn dagelijkse activiteiten |
| Dagelijkse activiteit (bijv. werk, studie, huishouden, gezins- en vrijetijdsactiviteiten) | Ik heb ***een beetje*** problemen met mijn dagelijkse activiteiten |
| Dagelijkse activiteit (bijv. werk, studie, huishouden, gezins- en vrijetijdsactiviteiten) | Ik heb ***matige*** problemen met mijn dagelijkse activiteiten |
| Dagelijkse activiteit (bijv. werk, studie, huishouden, gezins- en vrijetijdsactiviteiten) | Ik heb ***ernstige*** problemen met mijn dagelijkse activiteiten |
| Dagelijkse activiteit (bijv. werk, studie, huishouden, gezins- en vrijetijdsactiviteiten) | Ik ben ***niet*** in staat mijn dagelijkse activiteiten uit te voeren |
| Pijn / Ongemak | Ik heb ***geen*** pijn of ongemak |
| Pijn / Ongemak | Ik heb ***een beetje*** pijn of ongemak |
| Pijn / Ongemak | Ik heb ***matige*** pijn of ongemak |
| Pijn / Ongemak | Ik heb ***ernstige*** pijn of ongemak |
| Pijn / Ongemak | Ik heb ***extreem*** pijn of ongemak |
| Angst / Somberheid | Ik ben ***niet*** angstig of somber |
| Angst / Somberheid | Ik ben ***een beetje*** angstig of somber |
| Angst / Somberheid | Ik ben ***matig*** angstig of somber |
| Angst / Somberheid | Ik ben ***erg*** angstig of somber |
| Angst / Somberheid | Ik ben ***extreem*** angstig of somber |

**EQ-5D-Y-5L (youth) version**

| **Domain header** | **Levels** |
| --- | --- |
| Beweging (lopen) | Ik heb ***geen*** problemen met lopen |
| Beweging (lopen) | Ik heb ***een beetje*** problemen met lopen |
| Beweging (lopen) | Ik heb ***wel wat*** problemen met lopen |
| Beweging (lopen) | Ik heb ***veel*** problemen met lopen |
| Beweging (lopen) | Ik kan ***niet*** lopen. |
| Voor mezelf zorgen | Ik heb ***geen*** problemen met mijzelf wassen of aankleden |
| Voor mezelf zorgen | Ik heb ***een beetje*** problemen met mijzelf wassen of aankleden |
| Voor mezelf zorgen | Ik heb ***wel wat*** problemen met mijzelf wassen of aankleden |
| Voor mezelf zorgen | Ik heb ***veel*** problemen met mijzelf wassen of aankleden |
| Voor mezelf zorgen | Ik kan mijzelf ***niet*** wassen of aankleden |
| Dagelijkse activiteiten (bijvoorbeeld naar school gaan, hobby’s, sporten, spelen, naar familie of vrienden gaan) | Ik heb ***geen*** problemen met mijn dagelijkse activiteiten |
| Dagelijkse activiteiten (bijvoorbeeld naar school gaan, hobby’s, sporten, spelen, naar familie of vrienden gaan) | Ik heb ***een beetje*** problemen met mijn dagelijkse activiteiten |
| Dagelijkse activiteiten (bijvoorbeeld naar school gaan, hobby’s, sporten, spelen, naar familie of vrienden gaan) | Ik heb ***wel wat*** problemen met mijn dagelijkse activiteiten |
| Dagelijkse activiteiten (bijvoorbeeld naar school gaan, hobby’s, sporten, spelen, naar familie of vrienden gaan) | Ik heb ***veel*** problemen met mijn dagelijkse activiteiten |
| Dagelijkse activiteiten (bijvoorbeeld naar school gaan, hobby’s, sporten, spelen, naar familie of vrienden gaan) | Ik kan mijn dagelijkse activiteiten ***niet*** doen |
| Pijn of andere klachten | Ik heb ***geen*** pijn of andere klachten |
| Pijn of andere klachten | Ik heb ***een beetje*** pijn of andere klachten |
| Pijn of andere klachten | Ik heb ***wel wat*** pijn of andere klachten |
| Pijn of andere klachten | Ik heb ***veel*** pijn of andere klachten |
| Pijn of andere klachten | Ik heb ***extreme*** pijn of andere klachten |
| Bezorgd, verdrietig of ongelukkig | Ik ben ***niet*** bezorgd, verdrietig of ongelukkig |
| Bezorgd, verdrietig of ongelukkig | Ik ben ***een beetje*** bezorgd, verdrietig of ongelukkig |
| Bezorgd, verdrietig of ongelukkig | Ik ben ***wel wat*** bezorgd, verdrietig of ongelukkig |
| Bezorgd, verdrietig of ongelukkig | Ik ben ***erg*** bezorgd, verdrietig of ongelukkig |
| Bezorgd, verdrietig of ongelukkig | Ik ben ***extreem*** bezorgd, verdrietig of ongelukkig |

**EQ (adult) VAS**

We willen weten hoe goed of slecht uw gezondheid VANDAAG is.

Deze meetschaal loopt van 0 tot 100.

100 staat voor de beste gezondheid die u zich kunt voorstellen. 0 staat voor de slechtste gezondheid die u zich kunt voorstellen.

Klik op de meetschaal om aan te geven hoe uw gezondheid VANDAAG is.

UW GEZONDHEID VANDAAG

[vertical scale 0-100]

**EQ (youth) VAS**

We willen graag weten hoe goed of slecht je gezondheid VANDAAG is.

Deze lijn gaat van 0 tot en met 100.

100 betekent de beste gezondheid die je je kunt voorstellen.

0 betekent de slechtste gezondheid die je je kunt voorstellen.

Klik op de lijn om aan te geven hoe goed of slecht je gezondheid is VANDAAG.

HOE GOED IS JE GEZONDHEID VANDAAG?

[vertical scale 0-100]
