# Supplementary material for: A head-to-head comparison of the adult EQ-5D-5L and youth EQ-5D-Y-5L in adolescents with idiopathic scoliosis
Source: J Patient Rep Outcomes. 2025 Jan 29;9:13. doi: 10.1186/s41687-025-00842-z (PMC11780234; doi:10.1186/s41687-025-00842-z)
Supplement: Supplementary file 2 — Supplementary Material 2 [file 41687_2025_842_MOESM2_ESM.docx]

**Supplemental Material 2: R packages used**

This document shows which R packages were used, their version, for what purpose, and references using digital object identifier (DOI) links if available. Additionally attached or loaded packages were not included.

R software: R version 4.3.1
Reference: R Core Team (2024). _R: A Language and Environment for Statistical Computing_. R Foundation for Statistical Computing, Vienna, Austria. <https://www.R-project.org/>.

For all analyses existing packages were used, except for Bland-Altman plots. Although these plots were created using ggplot2, input values were calculated using self-developed code. The difference was first calculated for each patient, and the mean and standard deviation (SD) was obtained. Then, Limits of Agreement were calculated as +/- 1.96 x the SD of the mean difference. 95% CIs were calculated using the Student T distribution surrounding these estimates.

| Base packages | Used for | DOI Reference |
| --- | --- | --- |
| stats | Spearman rank correlation coefficients | See above. |
| Installed |  |  |
| cocor_1.1-4 | Steiger test to compare ICC’s/kappa’s | 10.32614/CRAN.package.cocor |
| psych_2.4.6.26 | Kappa analysis | 10.32614/CRAN.package.psych |
| contingencytables_3.0.1 | Bowker test | 10.32614/CRAN.package.contingencytables |
| exact2x2_1.6.9 | McNemar test | 10.32614/CRAN.package.exact2x2 |
| tableone_0.13.2 | Creating tables | 10.32614/CRAN.package.tableone |
| irr_0.84.1 | Intraclass Correlation Coefficient analysis | 10.32614/CRAN.package.irr |
| writexl_1.5.0 | Data management | 10.32614/CRAN.package.writexl |
| readxl_1.4.3 | Data management | 10.32614/CRAN.package.readxl |
| dplyr_1.1.4 | Data management | 10.32614/CRAN.package.dplyr |
| ggplot2_3.5.1 | Creating figures | 10.32614/CRAN.package.ggplot2 |
| tidyverse_2.0.0 | Data management | 10.32614/CRAN.package.tidyverse |
