# Supplementary material for: A head-to-head comparison of the adult EQ-5D-5L and youth EQ-5D-Y-5L in adolescents with idiopathic scoliosis
Source: J Patient Rep Outcomes. 2025 Jan 29;9:13. doi: 10.1186/s41687-025-00842-z (PMC11780234; doi:10.1186/s41687-025-00842-z)

**Supplemental Material 3, Figures 1–4: Bland-Altman plots for test-retest reliability**

The y-axis depicts the difference between the intra-individual measurement of the EQ-5D-5L and EQ-5D-Y-5L. The x-axis depicts the average of these two measurements. Patients with complete baseline (first) and follow-up (second) measurements were included (about 75% of patients, n=79). The dashed lines indicate the mean difference between instruments and 95% limits of agreement. The red dotted lines represent the 95% confidence intervals for these estimates.

Abbreviations: LSS = level-sum-score (total of EQ-5D domains summed, ranging from 5 to 25); VAS = Visual Analogue Scale

Figure 1: Bland-Altman of the LSS of the EQ-5D-5L for first and second measurement.
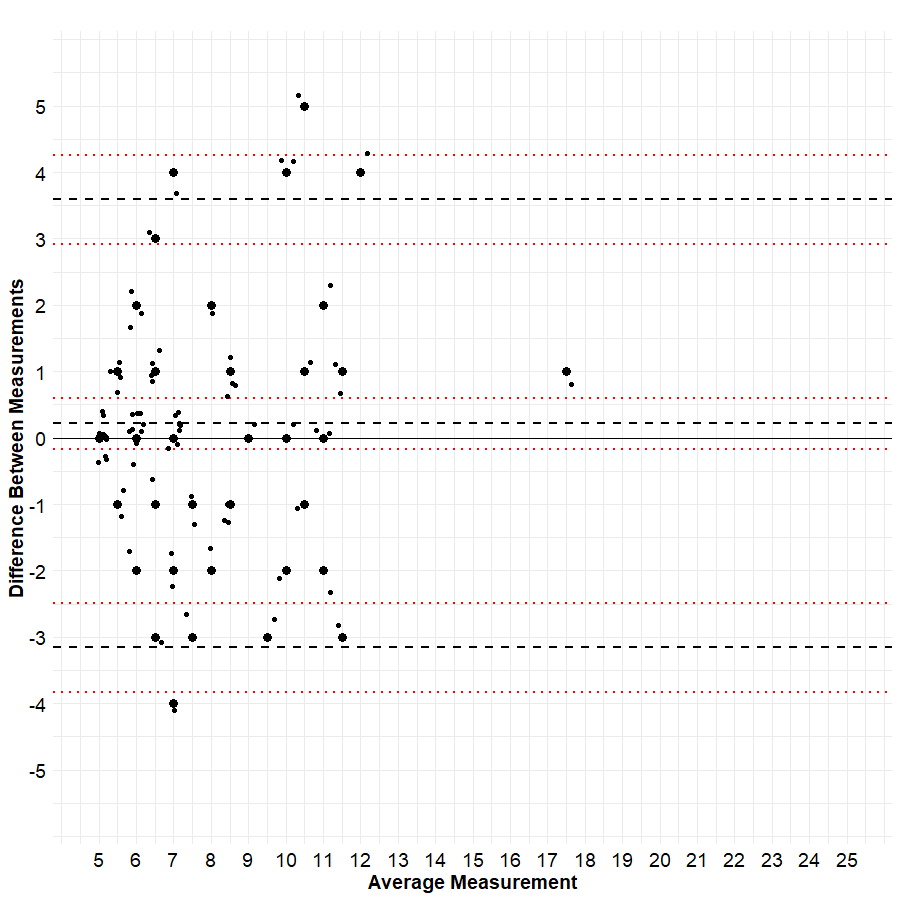


Figure 2: Bland-Altman of the LSS of the EQ-5D-Y-5L for first and second measurement.
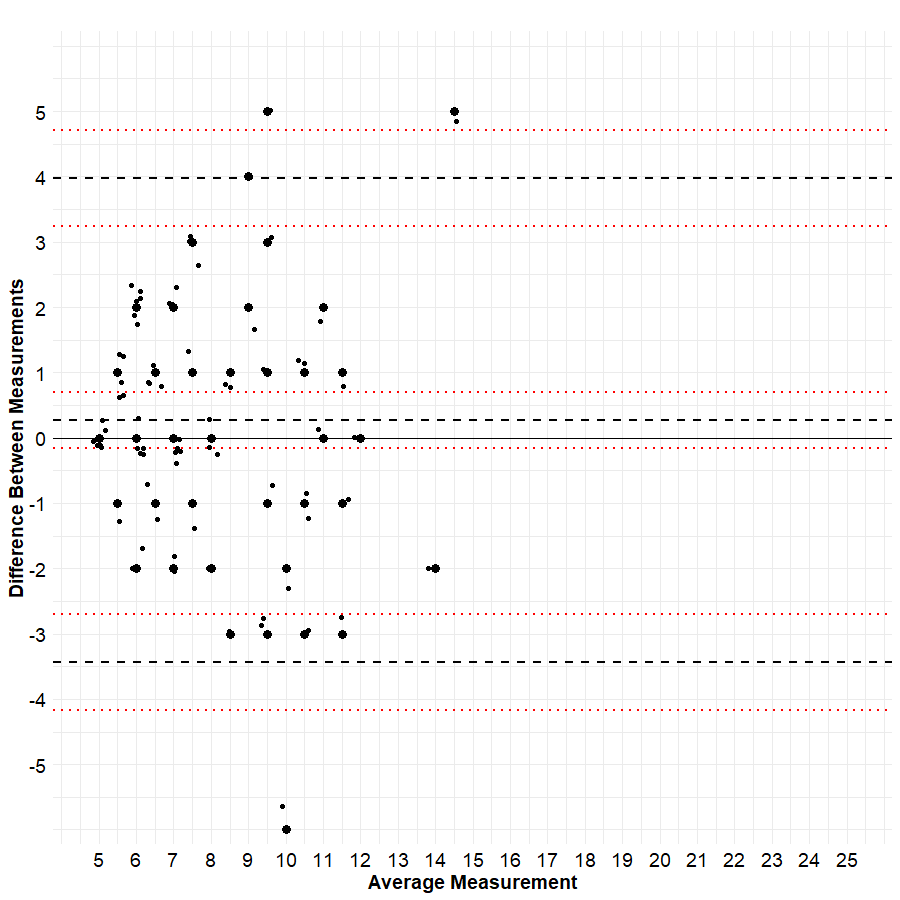


Figure 3: Bland-Altman of the VAS of the EQ-5D-5L for first and second measurement.
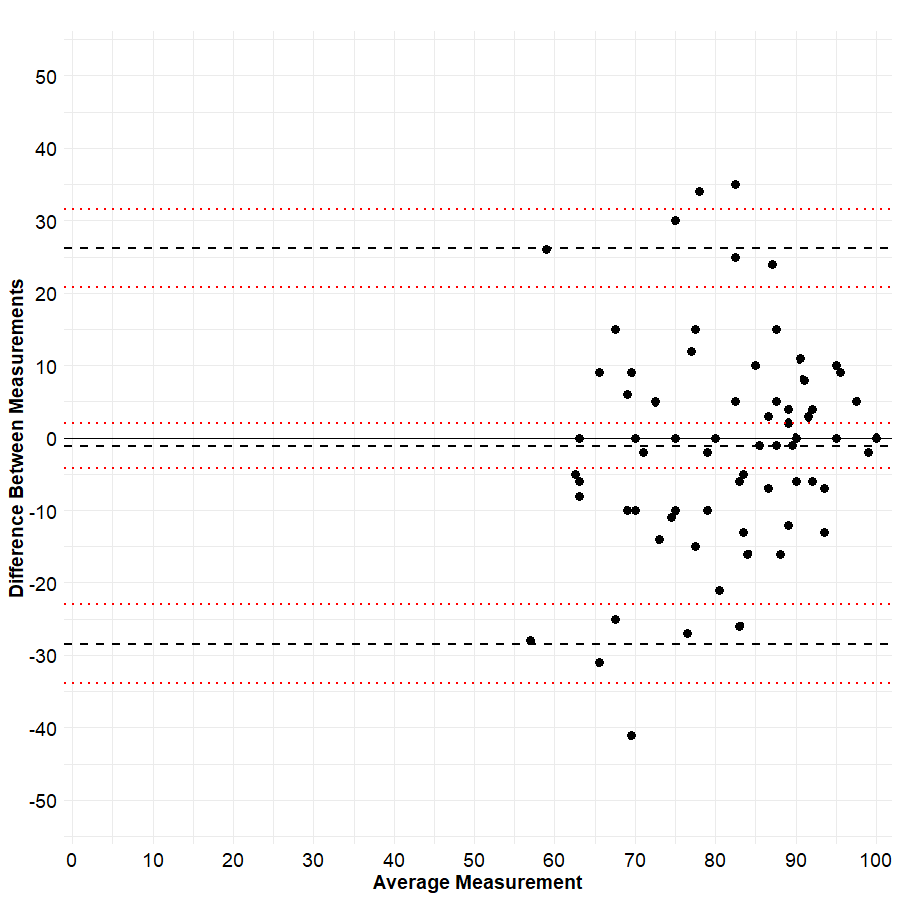


Figure 4: Bland-Altman of the VAS of the EQ-5D-Y-5L for first and second measurement.
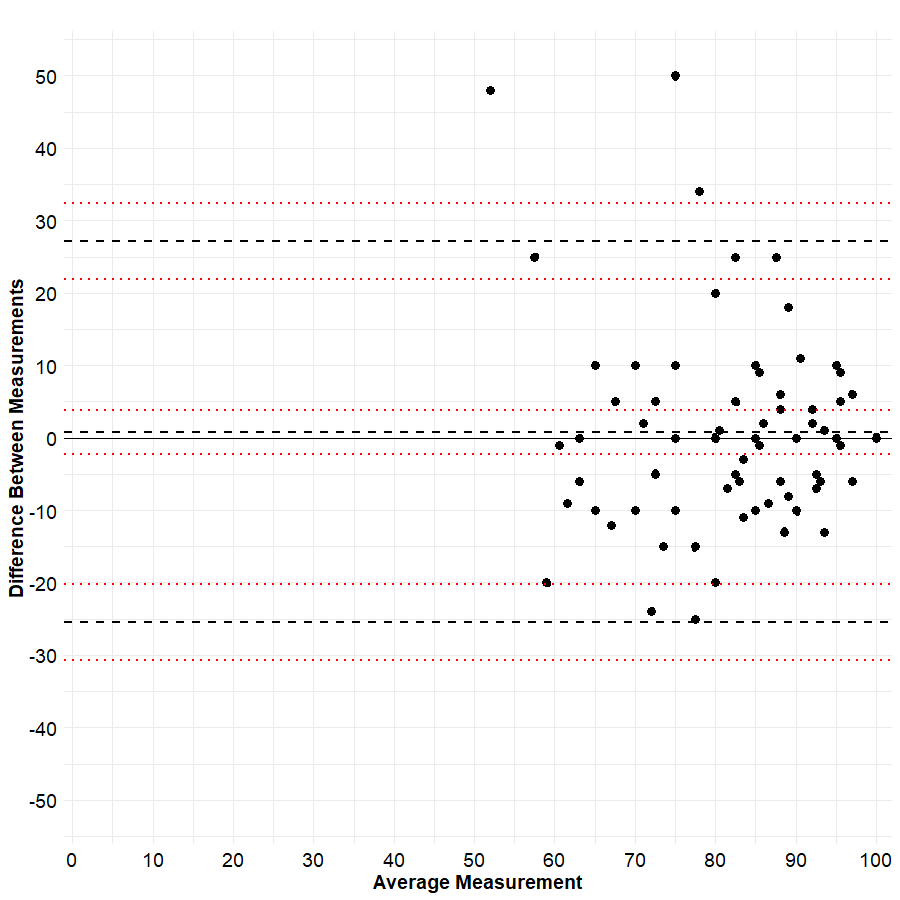

Supplement: Supplementary file 3 — Supplementary Material 3 [file 41687_2025_842_MOESM3_ESM.docx]
