# Supplementary material for: A head-to-head comparison of the adult EQ-5D-5L and youth EQ-5D-Y-5L in adolescents with idiopathic scoliosis
Source: J Patient Rep Outcomes. 2025 Jan 29;9:13. doi: 10.1186/s41687-025-00842-z (PMC11780234; doi:10.1186/s41687-025-00842-z)
Supplement: Supplementary file 4 — Supplementary Material 4 [file 41687_2025_842_MOESM4_ESM.docx]

**Supplemental Material 4, Tables 1–16: Sensitivity analysis**

**Overall**

- The cobb angle represents the severity of scoliosis disease measured on radiographs.
- SRS-22r is a scoliosis disease-specific questionnaire.
- Education was dichotomized into a practical and theoretical education; primary education was omitted from this subgroup comparison due to the low number of children in this group (n=8).

**Sensitivity analysis**

- Tables 1 through 8 depict summary statistics for and agreement between EQ-5D versions (EQ-5D-5L and EQ-5D-Y-5L) for subgroups based on cobb angle, SRS-22r, education and age.
- Tables 9 through 16 depict test-retest reliability for these same subgroups.
- For agreement and test-retest reliability, we used weighted (quadratic) Kappa and ICC analyse for domains and aggregate scores, respectively.

Abbreviations: MO = mobility, SC = self-care, UA = usual activities, PD = pain, AD = anxiety/depression, LSS = level sum score, VAS = visual analogue scale, SD = standard deviation, IQR = interquartile range, ICC = Intraclass Correlation Coefficient, 95% CI = 95% confidence interval.

Table 1: Summary statistics and agreement for patients with a cobb angle <30 (n=46)

|  | **EQ-5D-5L** | | | | | **EQ-5D-Y-5L** | | | | | **Agreement** | |
| --- | --- | --- | --- | --- | --- | --- | --- | --- | --- | --- | --- | --- |
|  | **Mean** | **SD** | **Median** | **IQR** | **Range** | **Mean** | **SD** | **Median** | **IQR** | **Range** |  |  |
| *Domain* |  |  |  |  |  |  |  |  |  |  | **Kappa** | **95% CI** |
| MO | 1.3 | 0.7 | 1 | 1 – 1 | 1 – 5 | 1.2 | 0.5 | 1 | 1 – 1 | 1 – 3 | 0.28 | -0.06 – 0.62 |
| SC | 1.3 | 0.6 | 1 | 1 – 1.8 | 1 – 3 | 1.3 | 0.5 | 1 | 1 – 1.75 | 1 – 3 | 0.72 | 0.46 – 0.98 |
| UA | 1.5 | 0.7 | 1 | 1 – 2 | 1 – 4 | 1.6 | 0.8 | 1 | 1 – 2 | 1 – 4 | 0.49 | 0.22 – 0.76 |
| PD | 1.8 | 0.7 | 2 | 1 – 2 | 1 – 3 | 1.7 | 0.7 | 2 | 1 – 2 | 1 – 4 | 0.56 | 0.30 – 0.81 |
| AD | 1.4 | 0.7 | 1 | 1 – 2 | 1 – 3 | 1.6 | 0.8 | 1 | 1 – 2 | 1 – 4 | 0.27 | -0.02 – 0.56 |
| *Aggregrate* |  |  |  |  |  |  |  |  |  |  | **ICC** | **95% CI** |
| LSS | 7.3 | 2.0 | 7 | 6 – 8 | 5 – 12 | 7.5 | 2.1 | 7 | 6 – 9 | 5 – 12 | 0.60 | 0.38 – 0.76 |
| VAS | 82.4 | 14.5 | 87 | 75 – 94 | 43 – 100 | 83.3 | 13.1 | 87 | 76.5 – 93.3 | 49 – 100 | 0.64 | 0.43 – 0.78 |

Table 2: Summary statistics and agreement for patients with a cobb angle >=30 (n=60)

|  | **EQ-5D-5L** | | | | | **EQ-5D-Y-5L** | | | | | **Agreement** | |
| --- | --- | --- | --- | --- | --- | --- | --- | --- | --- | --- | --- | --- |
|  | **Mean** | **SD** | **Median** | **IQR** | **Range** | **Mean** | **SD** | **Median** | **IQR** | **Range** |  |  |
| *Domain* |  |  |  |  |  |  |  |  |  |  | **Kappa** | **95% CI** |
| MO | 1.3 | 0.6 | 1 | 1 – 1 | 1 – 3 | 1.3 | 0.6 | 1 | 1 – 1 | 1 – 4 | 0.87 | 0.77 – 0.97 |
| SC | 1.2 | 0.4 | 1 | 1 – 1 | 1 – 3 | 1.2 | 0.4 | 1 | 1 – 1 | 1 – 3 | 0.79 | 0.56 – 1.00 |
| UA | 1.7 | 0.9 | 1 | 1 – 2 | 1 – 5 | 1.7 | 0.8 | 1.5 | 1 – 2 | 1 – 4 | 0.46 | 0.24 – 0.69 |
| PD | 2.1 | 0.9 | 2 | 1 – 3 | 1 – 4 | 2.0 | 0.9 | 2 | 1 – 2.3 | 1 – 4 | 0.74 | 0.61 – 0.87 |
| AD | 1.9 | 1.0 | 2 | 1 – 2 | 1 – 5 | 2.1 | 0.9 | 2 | 1 – 3 | 1 – 5 | 0.71 | 0.55 – 0.86 |
| *Aggregrate* |  |  |  |  |  |  |  |  |  |  | **ICC** | **95% CI** |
| LSS | 8.0 | 2.9 | 7 | 6 – 9 | 5 – 18 | 8.2 | 2.5 | 7.5 | 6.75 – 9.3 | 5 – 17 | 0.87 | 0.79 – 0.92 |
| VAS | 83.0 | 14.3 | 86 | 70 – 95.3 | 42 – 100 | 82.5 | 13.9 | 85 | 71 – 94.5 | 45 – 100 | 0.92 | 0.87 – 0.95 |

Table 3: Summary statistics and agreement for patients with a SRS-22r in range 1-4 (lower 50%) (n=55)

|  | **EQ-5D-5L** | | | | | **EQ-5D-Y-5L** | | | | | **Agreement** | |
| --- | --- | --- | --- | --- | --- | --- | --- | --- | --- | --- | --- | --- |
|  | **Mean** | **SD** | **Median** | **IQR** | **Range** | **Mean** | **SD** | **Median** | **IQR** | **Range** |  |  |
| *Domain* |  |  |  |  |  |  |  |  |  |  | **Kappa** | **95% CI** |
| MO | 1.4 | 0.6 | 1 | 1 – 2 | 1 – 3 | 1.3 | 0.6 | 1 | 1 – 2 | 1 – 4 | 0.70 | 0.46 – 0.93 |
| SC | 1.3 | 0.6 | 1 | 1 – 1 | 1 – 3 | 1.2 | 0.4 | 1 | 1 – 1 | 1 – 3 | 0.74 | 0.47 – 1.00 |
| UA | 2.0 | 0.9 | 2 | 1 – 2 | 1 – 5 | 1.9 | 0.8 | 2 | 1 – 2 | 1 – 4 | 0.53 | 0.34 – 0.72 |
| PD | 2.3 | 0.8 | 2 | 2 – 3 | 1 – 4 | 2.3 | 0.9 | 2 | 2 – 3 | 1 – 4 | 0.73 | 0.59 – 0.86 |
| AD | 2.1 | 1.0 | 2 | 1 – 3 | 1 – 5 | 2.3 | 0.9 | 2 | 2 – 3 | 1 – 5 | 0.55 | 0.33 – 0.77 |
| *Aggregrate* |  |  |  |  |  |  |  |  |  |  | **ICC** | **95% CI** |
| LSS | 9.0 | 2.8 | 8 | 7 – 10.5 | 5 – 18 | 9.0 | 2.4 | 9 | 7 – 10.5 | 5 – 17 | 0.81 | 0.70 – 0.89 |
| VAS | 75.2 | 14.6 | 72 | 67 – 88 | 42 – 100 | 77.0 | 14.0 | 75 | 69 – 88.5 | 45 – 100 | 0.71 | 0.55 – 0.82 |

Table 4: Summary statistics and agreement for patients with a SRS-22r in range 4-5 (upper 50%) (n=52)

|  | **EQ-5D-5L** | | | | | **EQ-5D-Y-5L** | | | | | **Agreement** | |
| --- | --- | --- | --- | --- | --- | --- | --- | --- | --- | --- | --- | --- |
|  | **Mean** | **SD** | **Median** | **IQR** | **Range** | **Mean** | **SD** | **Median** | **IQR** | **Range** |  |  |
| *Domain* |  |  |  |  |  |  |  |  |  |  | **Kappa** | **95% CI** |
| MO | 1.2 | 0.7 | 1 | 1 – 1 | 1 – 5 | 1.2 | 0.4 | 1 | 1 – 1 | 1 – 3 | 0.46 | 0.04 – 0.88 |
| SC | 1.2 | 0.4 | 1 | 1 – 1 | 1 – 2 | 1.2 | 0.5 | 1 | 1 – 1 | 1 – 3 | 0.78 | 0.60 – 0.96 |
| UA | 1.2 | 0.4 | 1 | 1 – 1 | 1 – 2 | 1.4 | 0.7 | 1 | 1 – 2 | 1 – 4 | 0.12 | -0.12 – 0.37 |
| PD | 1.6 | 0.6 | 2 | 1 – 2 | 1 – 3 | 1.5 | 0.5 | 1 | 1 – 2 | 1 – 3 | 0.34 | 0.06 – 0.63 |
| AD | 1.2 | 0.4 | 1 | 1 – 1 | 1 – 3 | 1.4 | 0.5 | 1 | 1 – 2 | 1 – 3 | 0.06 | -0.23 – 0.34 |
| *Aggregrate* |  |  |  |  |  |  |  |  |  |  | **ICC** | **95% CI** |
| LSS | 6.4 | 1.4 | 6 | 5 – 7 | 5 – 10 | 6.6 | 1.5 | 6 | 6 – 7 | 5 – 12 | 0.36 | 0.10 – 0.58 |
| VAS | 90.7 | 8.5 | 91 | 86.5 – 98.5 | 70 – 100 | 89.2 | 9.2 | 90 | 83.5 – 97 | 60 – 100 | 0.80 | 0.68 – 0.88 |

Table 5: Summary statistics and agreement for patients with a practical education (n=42)

|  | **EQ-5D-5L** | | | | | **EQ-5D-Y-5L** | | | | | **Agreement** | |
| --- | --- | --- | --- | --- | --- | --- | --- | --- | --- | --- | --- | --- |
|  | **Mean** | **SD** | **Median** | **IQR** | **Range** | **Mean** | **SD** | **Median** | **IQR** | **Range** |  |  |
| *Domain* |  |  |  |  |  |  |  |  |  |  | **Kappa** | **95% CI** |
| MO | 1.3 | 0.7 | 1 | 1 – 1 | 1 – 5 | 1.2 | 0.6 | 1 | 1 – 1 | 1 – 4 | 0.42 | -0.09 – 0.92 |
| SC | 1.1 | 0.3 | 1 | 1 – 1 | 1 – 2 | 1.1 | 0.3 | 1 | 1 – 1 | 1 – 2 | 0.63 | 0.24 – 1.00 |
| UA | 1.5 | 0.8 | 1 | 1 – 2 | 1 – 4 | 1.6 | 0.8 | 1 | 1 – 2 | 1 – 4 | 0.62 | 0.37 – 0.87 |
| PD | 2.0 | 0.8 | 2 | 1 – 2.75 | 1 – 4 | 1.9 | 0.9 | 2 | 1 – 2 | 1 – 4 | 0.54 | 0.33 – 0.75 |
| AD | 1.5 | 0.9 | 1 | 1 – 2 | 1 – 4 | 1.7 | 0.8 | 2 | 1 – 2 | 1 – 4 | 0.50 | 0.25 – 0.76 |
| *Aggregrate* |  |  |  |  |  |  |  |  |  |  | **ICC** | **95% CI** |
| LSS | 7.5 | 2.5 | 7 | 5.25 – 9 | 5 – 14 | 7.5 | 2.2 | 7 | 6 – 8 | 5 – 13 | 0.80 | 0.66 – 0.89 |
| VAS | 85.4 | 14.5 | 90 | 74.75 – 98 | 43 – 100 | 85.2 | 14.7 | 90 | 76.5 – 99.5 | 49 – 100 | 0.93 | 0.88 – 0.96 |

Table 6: Summary statistics and agreement for patients with a theoretical education (n=57)

|  | **EQ-5D-5L** | | | | | **EQ-5D-Y-5L** | | | | | **Agreement** | |
| --- | --- | --- | --- | --- | --- | --- | --- | --- | --- | --- | --- | --- |
|  | **Mean** | **SD** | **Median** | **IQR** | **Range** | **Mean** | **SD** | **Median** | **IQR** | **Range** |  |  |
| *Domain* |  |  |  |  |  |  |  |  |  |  | **Kappa** | **95% CI** |
| MO | 1.3 | 0.5 | 1 | 1 – 1 | 1 – 3 | 1.3 | 0.5 | 1 | 1 – 1 | 1 – 3 | 0.84 | 0.71 – 0.97 |
| SC | 1.2 | 0.5 | 1 | 1 – 1 | 1 – 3 | 1.3 | 0.5 | 1 | 1 – 2 | 1 – 3 | 0.90 | 0.78 – 1.00 |
| UA | 1.7 | 0.8 | 2 | 1 – 2 | 1 – 5 | 1.7 | 0.8 | 2 | 1 – 2 | 1 – 4 | 0.34 | 0.13 – 0.55 |
| PD | 2.0 | 0.9 | 2 | 1 – 2 | 1 – 4 | 1.9 | 0.9 | 2 | 1 – 2 | 1 – 4 | 0.81 | 0.68 – 0.94 |
| AD | 1.7 | 0.9 | 1 | 1 – 2 | 1 – 5 | 1.9 | 1.0 | 2 | 1 – 2 | 1 – 5 | 0.68 | 0.49 – 0.87 |
| *Aggregrate* |  |  |  |  |  |  |  |  |  |  | **ICC** | **95% CI** |
| LSS | 7.9 | 2.7 | 7 | 6 – 9 | 5 – 18 | 8.1 | 2.4 | 8 | 6 – 9 | 5 – 17 | 0.82 | 0.71 – 0.89 |
| VAS | 80.3 | 14.0 | 81 | 70 – 90 | 42 – 100 | 80.4 | 12.2 | 81 | 70 – 90 | 45 – 100 | 0.64 | 0.45 – 0.77 |

Table 7: Summary statistics and agreement for patients with an age in range 12-14 (lower 50% ) (n=65)

|  | **EQ-5D-5L** | | | | | **EQ-5D-Y-5L** | | | | | **Agreement** | |
| --- | --- | --- | --- | --- | --- | --- | --- | --- | --- | --- | --- | --- |
|  | **Mean** | **SD** | **Median** | **IQR** | **Range** | **Mean** | **SD** | **Median** | **IQR** | **Range** |  |  |
| *Domain* |  |  |  |  |  |  |  |  |  |  | **Kappa** | **95% CI** |
| MO | 1.3 | 0.7 | 1 | 1 – 1 | 1 – 5 | 1.3 | 0.6 | 1 | 1 – 2 | 1 – 4 | 0.56 | 0.16 – 0.96 |
| SC | 1.2 | 0.5 | 1 | 1 – 1 | 1 – 3 | 1.2 | 0.5 | 1 | 1 – 1 | 1 – 3 | 0.79 | 0.65 – 0.92 |
| UA | 1.6 | 0.7 | 1 | 1 – 2 | 1 – 4 | 1.6 | 0.8 | 1 | 1 – 2 | 1 – 4 | 0.48 | 0.32 – 0.64 |
| PD | 2.0 | 0.9 | 2 | 1 – 2 | 1 – 4 | 2.0 | 0.9 | 2 | 1 – 2 | 1 – 4 | 0.72 | 0.59 – 0.86 |
| AD | 1.7 | 1.0 | 1 | 1 – 2 | 1 – 5 | 1.9 | 0.9 | 2 | 1 – 2 | 1 – 5 | 0.64 | 0.44 – 0.83 |
| *Aggregrate* |  |  |  |  |  |  |  |  |  |  | **ICC** | **95% CI** |
| LSS | 7.9 | 2.6 | 7 | 6 – 10 | 5 – 18 | 8.1 | 2.4 | 8 | 6 – 9 | 5 – 17 | 0.82 | 0.72 – 0.89 |
| VAS | 82.7 | 14.0 | 85 | 72 – 95 | 42 – 100 | 83.2 | 13.3 | 85 | 75 – 95 | 45 – 100 | 0.81 | 0.70 – 0.88 |

Table 8: Summary statistics and agreement for patients with age in range 15-17 (upper 50%) (n=42)

|  | **EQ-5D-5L** | | | | | **EQ-5D-Y-5L** | | | | | **Agreement** | |
| --- | --- | --- | --- | --- | --- | --- | --- | --- | --- | --- | --- | --- |
|  | **Mean** | **SD** | **Median** | **IQR** | **Range** | **Mean** | **SD** | **Median** | **IQR** | **Range** |  |  |
| *Domain* |  |  |  |  |  |  |  |  |  |  | **Kappa** | **95% CI** |
| MO | 1.2 | 0.5 | 1 | 1 – 1 | 1 – 3 | 1.1 | 0.4 | 1 | 1 – 1 | 1 – 2 | 0.53 | 0.19 – 0.88 |
| SC | 1.2 | 0.5 | 1 | 1 – 1 | 1 – 3 | 1.1 | 0.4 | 1 | 1 – 1 | 1 – 3 | 0.71 | 0.27 – 1.00 |
| UA | 1.6 | 0.9 | 1 | 1 – 2 | 1 – 5 | 1.7 | 0.8 | 1.5 | 1 – 2 | 1 – 4 | 0.47 | 0.17 – 0.78 |
| PD | 1.9 | 0.8 | 2 | 1 – 2 | 1 – 4 | 1.7 | 0.8 | 1.5 | 1 – 2 | 1 – 4 | 0.61 | 0.36 – 0.86 |
| AD | 1.5 | 0.8 | 1 | 1 – 2 | 1 – 4 | 1.8 | 0.8 | 2 | 1 – 2 | 1 – 4 | 0.52 | 0.28 – 0.76 |
| *Aggregrate* |  |  |  |  |  |  |  |  |  |  | **ICC** | **95% CI** |
| LSS | 7.4 | 2.6 | 7 | 6 – 8 | 5 – 18 | 7.5 | 2.2 | 7 | 6 – 8.8 | 5 – 14 | 0.73 | 0.55 – 0.85 |
| VAS | 82.8 | 14.8 | 87 | 70 – 95 | 43 – 100 | 82.4 | 13.8 | 87 | 70 – 93 | 49 – 100 | 0.79 | 0.64 – 0.88 |

Table 9: Test-retest reliability in patients with a cobbs angle <30 (n=32)

|  | **EQ-5D-5L** | | **EQ-5D-Y-5L** | |
| --- | --- | --- | --- | --- |
| *Domain* | **Kappa** | **95% CI** | **Kappa** | **95% CI** |
| MO | 0.33 | -0.02 – 0.69 | 0.64 | 0.18 – 1.00 |
| SC | 0.10 | -0.17 – 0.37 | 0.23 | -0.16 – 0.61 |
| UA | 0.85 | 0.70 – 1.00 | 0.58 | 0.33 – 0.84 |
| PD | 0.61 | 0.42 – 0.81 | 0.74 | 0.53 – 0.95 |
| AD | 0.64 | 0.39 – 0.88 | 0.53 | 0.25 – 0.80 |
| *Aggregrate* | **ICC** | **95% CI** | **ICC** | **95% CI** |
| LSS | 0.78 | 0.60 – 0.89 | 0.70 | 0.47 – 0.84 |
| VAS | 0.41 | 0.08 – 0.66 | 0.54 | 0.24 – 0.75 |

Table 10: Test-retest reliability in patients with a cobbs angle >=30 (n=45)

|  | **EQ-5D-5L** | | **EQ-5D-Y-5L** | |
| --- | --- | --- | --- | --- |
| *Domain* | **Kappa** | **95% CI** | **Kappa** | **95% CI** |
| MO | 0.42 | 0.16 – 0.68 | 0.44 | 0.22 – 0.65 |
| SC | 0.52 | 0.23 – 0.82 | 0.13 | -0.18 – 0.45 |
| UA | 0.49 | 0.27 – 0.71 | 0.45 | 0.19 – 0.72 |
| PD | 0.66 | 0.49 – 0.83 | 0.50 | 0.27 – 0.73 |
| AD | 0.66 | 0.42 – 0.89 | 0.74 | 0.60 – 0.88 |
| *Aggregrate* | **ICC** | **95% CI** | **ICC** | **95% CI** |
| LSS | 0.74 | 0.57 – 0.85 | 0.67 | 0.47 – 0.80 |
| VAS | 0.50 | 0.24 – 0.69 | 0.49 | 0.23 – 0.68 |

Table 11: Test-retest reliability in patients with a SRS-22r in range 1-4 (lower 50%) (n=55)

|  | **EQ-5D-5L** | | **EQ-5D-Y-5L** | |
| --- | --- | --- | --- | --- |
| *Domain* | **Kappa** | **95% CI** | **Kappa** | **95% CI** |
| MO | 0.26 | -0.01 – 0.53 | 0.40 | 0.17 – 0.64 |
| SC | 0.21 | -0.11 – 0.54 | 0.19 | -0.12 – 0.51 |
| UA | 0.48 | 0.19 – 0.76 | 0.52 | 0.30 – 0.74 |
| PD | 0.59 | 0.39 – 0.79 | 0.45 | 0.20 – 0.70 |
| AD | 0.60 | 0.34 – 0.85 | 0.70 | 0.56 – 0.84 |
| *Aggregrate* | **ICC** | **95% CI** | **ICC** | **95% CI** |
| LSS | 0.65 | 0.43 – 0.79 | 0.54 | 0.28 – 0.73 |
| VAS | 0.28 | -0.01 – 0.53 | 0.49 | 0.21 – 0.69 |

Table 12: Test-retest reliability in patients with a SRS-22r in range 4-5 (upper 50%) (n=52)

|  | **EQ-5D-5L** | | **EQ-5D-Y-5L** | |
| --- | --- | --- | --- | --- |
| *Domain* | **Kappa** | **95% CI** | **Kappa** | **95% CI** |
| MO | 0.47 | 0.03 – 0.91 | 0.55 | 0.15 – 0.96 |
| SC | 0.45 | 0.00 – 0.89 | 0.12 | -0.27 – 0.51 |
| UA | 0.70 | 0.47 – 0.93 | 0.10 | -0.12 – 0.32 |
| PD | 0.43 | 0.17 – 0.69 | 0.47 | 0.22 – 0.73 |
| AD | 0.21 | -0.18 – 0.59 | -0.06 | -0.31 – 0.18 |
| *Aggregrate* | **ICC** | **95% CI** | **ICC** | **95% CI** |
| LSS | 0.42 | 0.11 – 0.65 | 0.24 | -0.06 – 0.51 |
| VAS | 0.38 | 0.09 – 0.62 | 0.32 | 0.01 – 0.58 |

Table 13: Test-retest reliability in patients with a practical education (n=31)

|  | **EQ-5D-5L** | | **EQ-5D-Y-5L** | |
| --- | --- | --- | --- | --- |
| *Domain* | **Kappa** | **95% CI** | **Kappa** | **95% CI** |
| MO | 0.31 | -0.12 – 0.75 | 0.35 | -0.10 – 0.80 |
| SC | 0.43 | -0.02 – 0.89 | -0.09 | -0.19 – 0.00 |
| UA | 0.71 | 0.46 – 0.96 | 0.64 | 0.43 – 0.86 |
| PD | 0.75 | 0.63 – 0.87 | 0.71 | 0.54 – 0.87 |
| AD | 0.71 | 0.48 – 0.94 | 0.76 | 0.62 – 0.90 |
| *Aggregrate* | **ICC** | **95% CI** | **ICC** | **95% CI** |
| LSS | 0.81 | 0.65 – 0.91 | 0.81 | 0.64 – 0.90 |
| VAS | 0.54 | 0.22 – 0.75 | 0.42 | 0.09 – 0.67 |

Table 14: Test-retest reliability in patients with a theoretical education (n=40)

|  | **EQ-5D-5L** | | **EQ-5D-Y-5L** | |
| --- | --- | --- | --- | --- |
| *Domain* | **Kappa** | **95% CI** | **Kappa** | **95% CI** |
| MO | 0.42 | 0.18 – 0.65 | 0.57 | 0.39 – 0.75 |
| SC | 0.42 | 0.11 – 0.73 | 0.23 | -0.10 – 0.56 |
| UA | 0.69 | 0.54 – 0.84 | 0.40 | 0.12 – 0.69 |
| PD | 0.59 | 0.36 – 0.81 | 0.50 | 0.23 – 0.77 |
| AD | 0.63 | 0.31 – 0.95 | 0.66 | 0.45 – 0.87 |
| *Aggregrate* | **ICC** | **95% CI** | **ICC** | **95% CI** |
| LSS | 0.75 | 0.59 – 0.86 | 0.62 | 0.39 – 0.78 |
| VAS | 0.34 | 0.04 – 0.58 | 0.59 | 0.35 – 0.76 |

Table 15: Test-retest reliability in patients with an age in range 12-14 (lower 50%) (n=47)

|  | **EQ-5D-5L** | | **EQ-5D-Y-5L** | |
| --- | --- | --- | --- | --- |
| *Domain* | **Kappa** | **95% CI** | **Kappa** | **95% CI** |
| MO | 0.40 | 0.13 – 0.67 | 0.47 | 0.24 – 0.70 |
| SC | 0.36 | 0.05 – 0.68 | 0.17 | -0.12 – 0.46 |
| UA | 0.57 | 0.36 – 0.77 | 0.57 | 0.35 – 0.78 |
| PD | 0.62 | 0.44 – 0.80 | 0.46 | 0.21 – 0.70 |
| AD | 0.61 | 0.35 – 0.86 | 0.70 | 0.54 – 0.87 |
| *Aggregrate* | **ICC** | **95% CI** | **ICC** | **95% CI** |
| LSS | 0.70 | 0.52 – 0.82 | 0.65 | 0.45 – 0.79 |
| VAS | 0.52 | 0.29 – 0.70 | 0.61 | 0.40 – 0.77 |

Table 16: Test-retest reliability in patients with an age in range 15-17 (upper 50%) (n=31)

|  | **EQ-5D-5L** | | **EQ-5D-Y-5L** | |
| --- | --- | --- | --- | --- |
| *Domain* | **Kappa** | **95% CI** | **Kappa** | **95% CI** |
| MO | 0.39 | 0.07 – 0.71 | 0.55 | 0.24 – 0.87 |
| SC | 0.11 | -0.25 – 0.48 | 0.20 | -0.27 – 0.67 |
| UA | 0.73 | 0.46 – 1.00 | 0.44 | 0.12 – 0.76 |
| PD | 0.73 | 0.58 – 0.88 | 0.76 | 0.57 – 0.94 |
| AD | 0.82 | 0.66 – 0.97 | 0.66 | 0.48 – 0.83 |
| *Aggregrate* | **ICC** | **95% CI** | **ICC** | **95% CI** |
| LSS | 0.88 | 0.76 – 0.94 | 0.76 | 0.56 – 0.88 |
| VAS | 0.34 | -0.02 – 0.62 | 0.33 | -0.03 – 0.61 |
